# Cross-species analysis of gene expression in non-model mammals: reproducibility of hybridization on high density oligonucleotide microarrays

**DOI:** 10.1186/1471-2164-8-89

**Published:** 2007-04-03

**Authors:** Manuel Nieto-Díaz, Wolfgang Pita-Thomas, Manuel Nieto-Sampedro

**Affiliations:** 1 Neural Plasticity Department, Instituto de Neurobiología Ramón y Cajal (CSIC), Avda. Doctor Arce 37, 28002 Madrid, Spain; 2 Experimental Neurology Unit, Hospital Nacional de Parapléjicos (SESCAM), Ctra. La Peraleda s/n, 45071 Toledo, Spain

## Abstract

**Background:**

Gene expression profiles of non-model mammals may provide valuable data for biomedical and evolutionary studies. However, due to lack of sequence information of other species, DNA microarrays are currently restricted to humans and a few model species. This limitation may be overcome by using arrays developed for a given species to analyse gene expression in a related one, an approach known as "cross-species analysis". In spite of its potential usefulness, the accuracy and reproducibility of the gene expression measures obtained in this way are still open to doubt. The present study examines whether or not hybridization values from cross-species analyses are as reproducible as those from same-species analyses when using Affymetrix oligonucleotide microarrays.

**Results:**

The reproducibility of the probe data obtained hybridizing deer, Old-World primates, and human RNA samples to Affymetrix human GeneChip^® ^U133 Plus 2.0 was compared. The results show that cross-species hybridization affected neither the distribution of the hybridization reproducibility among different categories, nor the reproducibility values of the individual probes. Our analyses also show that a 0.5% of the probes analysed in the U133 plus 2.0 GeneChip are significantly associated to un-reproducible hybridizations. Such probes-called in the text un-reproducible probe sequences- do not increase in number in cross-species analyses.

**Conclusion:**

Our study demonstrates that cross-species analyses do not significantly affect hybridization reproducibility of GeneChips, at least within the range of the mammal species analysed here. The differences in reproducibility between same-species and cross-species analyses observed in previous studies were probably caused by the analytical methods used to calculate the gene expression measures. Together with previous observations on the accuracy of GeneChips for cross-species analysis, our analyses demonstrate that cross-species hybridizations may provide useful gene expression data. However, the reproducibility and accuracy of these measures largely depends on the use of appropriated algorithms to derive the gene expression data from the probe data. Also, the identification of probes associated to un-reproducible hybridizations-useless for gene expression analyses- in the studied GeneChip, stress the need of a re-evaluation of the probes' performance.

## Background

DNA microarray technology is a basic tool to measure genomewide changes in gene expression. Microarray analysis of gene expression in non-model mammals may provide very valuable data for biomedical [1-3] or evolutionary [4-7] studies. However, DNA microarrays are currently restricted to humans and a few model species, due to lack of sequence information for other species. This limitation could be overcome by using arrays developed for a given species to analyse gene expression in a related one [4,8-12]. This approach, known as "cross-species analysis", assumes that the RNA transcripts for one species will hybridize efficiently with the arrayed sequences of another species, provided that both species share enough sequence similarity (over 95% in orthologous 3'-UTR sequences according to Nagpal *et al*. [10]). The cross-species approach has been employed in several studies in mammals, using human microarrays to analyse closely related species, such as chimpanzees, orangutans and other primates [4], as well as more distantly related species, such as pigs, cows or dogs [8,9]. These studies assume that the short time of divergence between mammals (less that 100 million years) and the preservation of their protein function assures enough nucleotide-sequence conservation among species [9].

Among the existing DNA array platforms, Affymetrix high-density oligonucleotide GeneChips^® ^(Affymetrix, Santa Clara, CA, USA) have been repeatedly employed for cross-species analyses. GeneChips estimate gene expression measures-like the presence and abundance of a transcript- by applying analytical methods to the hybridization values of sets of 11 to 20 pairs of probes (probesets) for each transcript [13]. Each probe pair consists of a 25 bases long perfect match probe (PM), fully complementary to the target, and a 25 bases long mismatch probe (MM), that shares only 24 bases with the target sequence. The large number of probes *per *target used by Affymetrix microarrays represents an advantage for cross species analyses with respect to other microarray platforms, such as those based on cDNA probes. The presence of 11 to 20 probes *per *target increases the probability of having probes with enough sequence similarity with the target transcript to obtain a feasible measure of its expression [9]. In contrast, the long sequence probes in cDNA microarrays may favour the hybridization with orthologous genes from other species compared to the 25 bases long Affymetrix probes. Genechips also have the advantage of allowing worldwide researchers to access the same standardized arrays, the same sample processing methods, and the same image acquisition instruments to quantify gene expression.

Despite the potential usefulness of cross-species analyses, the quality of the gene expression measures obtained in this way is open to doubt. Two aspects of measurement quality appear as most important i) the accuracy of the measurement-the agreement between the observed and the true value of a measure; termed validity in statistical terminology-, and ii) the reproducibility (or precision; also called reliability in statistical jargon) of the measurement, i.e. whether repeated measurements will give similar values [14]. Different authors have examined both aspects of cross-species analyses using Affymetrix GeneChips, reaching diverse conclusions [9,10,15-17]. There is a general agreement in that the array sensitivity and, thus, the accuracy of the analysis, decreases with increasing sequence differences between the species being analyzed and the array species [9,15,16]. In a practical sense, this implies that cross-species analyses yield significantly more false negatives-genes that appear not to be expressed although they are really being expressed- than same-species analyses. This point was clearly illustrated by Chismar and co-workers [15] showing that the number of detected transcripts by a human GeneChip were a 50% lower when analysing *Macaca *samples than when analysing human samples. Accordingly, various authors have developed specific methods to correct the sensitivity reduction of cross-species analysis [9,10,16].

Data reproducibility has received less attention despite the fact that it is not possible to achieve accuracy in individual measurements if these measurements are associated to high variability. The percentage of transcripts that can be consistently detected as present (according to an Affymetrix algorithm [13]) across replicates is significantly reduced when *Macaca *RNA samples were hybridized to the Affymetrix Hu95Av5 human GeneChip, than when human RNA samples were hybridized to this chip [15]. A similar reduction in reproducibility of the present calls was observed by Wang *et al*. [17] when analysing *Macaca *and *Pan *samples with human GeneChips. In contrast, Dillman and Phillips [18] observed no reproducibility differences when the gene expression data generated by hybridizing human, *Chlorocebus *and *Macaca *RNA samples to the Affymetrix U133 plus 2.0 human GeneChip were compared, in agreement with previous observations from cross-species analyses using cDNA microarrays [8,19]. What is the real effect of cross-species analyses on the reproducibility of Affymetrix hybridization data? To explain these apparently contradictory results, we hypothesized that hybridization values were equally reproducible in cross-species and same-species analyses at least when restricted to mammals, and that contradictions arose from differences in the way gene expression measures were derived from the hybridization data of the Affymetrix probes.

To test these hypotheses, we have compared the reproducibility of hybridization data from different mammal samples analysed with the Affymetrix human GeneChip U133 Plus 2.0. Unlike previous studies that analysed reproducibility of gene expression measures like signal intensity or detection call, we have used probe intensity data. In this way, we strictly analysed hybridization reproducibility, leaving out the effect of the algorithms used to calculate the gene expression measures from the probe intensity values. We used specifically data from hybridization to GeneChip U133 Plus 2.0, to take advantage of the presence of probes with the same sequences in different probesets of this GeneChip. These internal replicas permit the study of reproducibility within the same chip, avoiding variations due to differences in the GeneChips or in their processing (including hybridization, staining and scanning), which may be responsible for much of the total variation in microarray analyses [20]. Probe intensity data originate from the analysis of human, three species of Old-World primates, and deer RNA samples. We compared data from such a differently related taxa to evaluate the effect of increasing sequence differences on hybridization reproducibility. Old World monkeys are closely related to humans (time of divergence 25–30 millions of years [21,22]) while deers (a representative of the Artiodactyla order) represent a more distantly related taxon, whose ancestors diverged from the ancestors of primates at the base of the Placental radiation, nearly 100 million of years ago [21,23]. Hybridization data from these species were used to test the following:

1. Whether cross-species hybridizations affected the distribution of the hybridization reproducibility, i.e., whether the number of sequences in different reproducibility categories was similar in same and cross-species analyses.

2. Whether there were probe sequences associated to irreproducible or poorly reproducible hybridizations, and whether cross-species analyses increased the proportion of these sequences with respect to same-species analyses.

3. Whether the reproducibility of each sequence tended to be lower in cross-species hybridizations than in same-species hybridizations.

To further test the effect of sequence differences on hybridization reproducibility, we repeated these three analyses comparing the hybridization data for repeated perfect match (PM) and mismatch (MM) sequences in the human samples. These comparisons permit to evaluate the effect of a known and fixed sequence difference (one change in the 13th base) on hybridization reproducibility. Finally, we studied the relationship between hybridization value and reproducibility, to test whether low hybridization values were less reproducible than high values and thus, whether cross-species analyses were less reproducible because they yielded lower hybridization signals than same-species analyses.

The results presented show that, within the range of the mammals studied, cross-species analyses do not significantly affect hybridization reproducibility and suggest that the analytical methods used to calculate the gene expression measures are responsible for the previously observed reproducibility differences between same-species and cross-species analyses. In parallel, we have identified several probe sequences associated to poorly reproducible hybridizations that should not to be taken into consideration for quantifying gene expression.

## Results

### Identification and characterization of the GeneChip repeated sequences

Affymetrix U133 Plus 2.0 GeneChip contains 15472 sequences (7736 PM and 7736 MM) repeated in at least two different probes. These sequences correspond to 17462 of the 604258 probe pairs present in this GeneChip. Twenty six of the repeated sequences (13 PM and 13 MM), corresponding to 52 probes (26 PM and 26 MM), hybridize to non-human RNA transcripts included in the array as spikes to test the array performance. The number of repetitions ranges from 2 to 20, with most sequences repeated just once (Table 1). A description of all repeated probes in the U133 plus 2.0 GeneChip-including the probeset to which the probe belongs, the oligonucleotide sequence, and the position of the probe in the GeneChip- is provided in the Additional file 1.

**Table 1:** Number of repeated probe pairs and sequences in the Affymetrix U133 Plus 2.0 GeneChip^®^.

Number of Repetitions	Number of Sequences	Number of Probe Pairs
2	12794	12794
3	1924	2886
4	462	924
5	174	435
6	74	222
7	22	77
8	8	32
9	4	18
11	2	11
12	2	12
15	2	15
16	2	16
20	2	20
**Total**	**15472**	**17462**

Both PM and MM sequences are considered to calculate the number of repeated sequences.

RNA samples used in all analyses are detailed in table 2. Hybridization values of each sample for all repeated sequences are provided in the Additional file 1. Normal distribution of these values was checked in 22 sequences with more than 6 repetitions, using Shapiro-Wilk W test (see Additional file 2). Hybridization values were normally distributed across replicas in most cases, though for some sequences differed significantly (p < 0.05) from the normal distribution, even after applying a Bonferroni correction. Non-normality in these cases was due to the presence of one or a few outlier probes with extreme values (see graphs in Additional file 2). No differences in normality were apparent between species or between PM and MM data. Kolmogorov Smirnoff and Lilliefors tests yielded comparable results (data not shown).

**Table 2:** Description of the data used in the present study.

**Code**	**Species**	**Data Origin**	**GEO database accession number**	**Sample description**
*DEER1*		Present study	GSM93225	Soft tissues of antler tip
*DEER2*	*Cervus*	Present study	GSM93226	Soft tissues of antler base
*DEER3*	*elaphus*	Present study	GSM93227	Soft tissues from skull frontal bone
*DEER4*		Present study	GSM93228	Soft tissues of antler tip

*CAET1*		Dillman and Phillips, 2005	GSM50690	Whole blood (see Dillman and Phillips, 2005)
*CAET2*	*Chlorocebus*	Dillman and Phillips, 2005	GSM50691	Whole blood (see Dillman and Phillips, 2005)
*CAET3*	*aethiops*	Dillman and Phillips, 2005	GSM50692	Whole blood (see Dillman and Phillips, 2005)
*CAET4*		Dillman and Phillips, 2005	GSM50693	Whole blood (see Dillman and Phillips, 2005)

*MFAS1*		Dillman and Phillips, 2005	GSM50694	Whole blood (see Dillman and Phillips, 2005)
*MFAS2*	*Macaca*	Dillman and Phillips, 2005	GSM50695	Whole blood (see Dillman and Phillips, 2005)
*MFAS3*	*fascicularis*	Dillman and Phillips, 2005	GSM50696	Whole blood (see Dillman and Phillips, 2005)
*MFAS4*		Dillman and Phillips, 2005	GSM50697	Whole blood (see Dillman and Phillips, 2005)

*HSAP1*		Dillman and Phillips, 2005	GSM50698	Whole blood (see Dillman and Phillips, 2005)
*HSAP2*	*Homo*	Dillman and Phillips, 2005	GSM50699	Whole blood (see Dillman and Phillips, 2005)
*HSAP3*	*sapiens*	Dillman and Phillips, 2005	GSM50700	Whole blood (see Dillman and Phillips, 2005)
*HSAP4*		Dillman and Phillips, 2005	GSM50701	Whole blood (see Dillman and Phillips, 2005)
*HSAP5*		Dillman and Phillips, 2005	GSM50702	Whole blood (see Dillman and Phillips, 2005)

*MMUL1*		Dillman and Phillips, 2005	GSM50703	Whole blood (see Dillman and Phillips, 2005)
*MMUL2*	*Macaca*	Dillman and Phillips, 2005	GSM50704	Whole blood (see Dillman and Phillips, 2005)
*MMUL3*	*mulatta*	Dillman and Phillips, 2005	GSM50705	Whole blood (see Dillman and Phillips, 2005)
*MMUL4*		Dillman and Phillips, 2005	GSM50706	Whole blood (see Dillman and Phillips, 2005)

Code corresponds to the code names for the samples. GEO database accession number detail the codes corresponding to the expression data in the GEO database [33]

### Effect of sequence differences on the distribution of hybridization variability

The coefficient of variation (CV) of the hybridization values for the repeated PM sequences in each sample for the various species was chosen as a measurement of hybridization reproducibility and used to analyse the hybridization reproducibility differences between same-species and cross-species analyses. Although within-subject standard deviation is the most commonly used measurement of reproducibility, it was rejected due to its significant correlation with the mean in all samples (see data in Additional file 3 and analysis in Additional file 4). A brief discussion justifying the use of CV to measure data reproducibility is provided in the methods section (fully discussed in [14,24,25]). CV data for all samples and sequences may be obtained from the Additional file 3.

**Figure 1:**
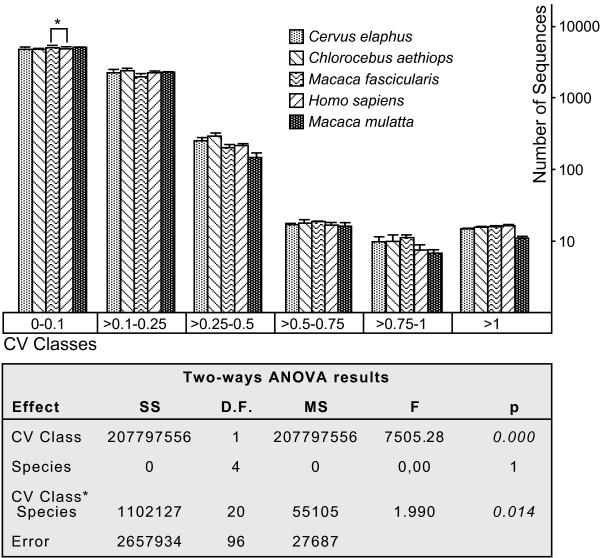
**Comparison of the number of sequences *per *CV class in the analysed species**. Each bar represents the mean number of PM sequences *per *class and species (error bar represents the Standard Error of the Mean). Below the graph there is a summary of the two-way ANOVA results, using the number of sequences as a dependent variable and CV classes and species as factors. The differences in the number of sequences *per *class between species are analyzed by the interaction term (CV Class*Species). Significant differences (p < 0.05) between species are marked by *.

**Figure 2:**
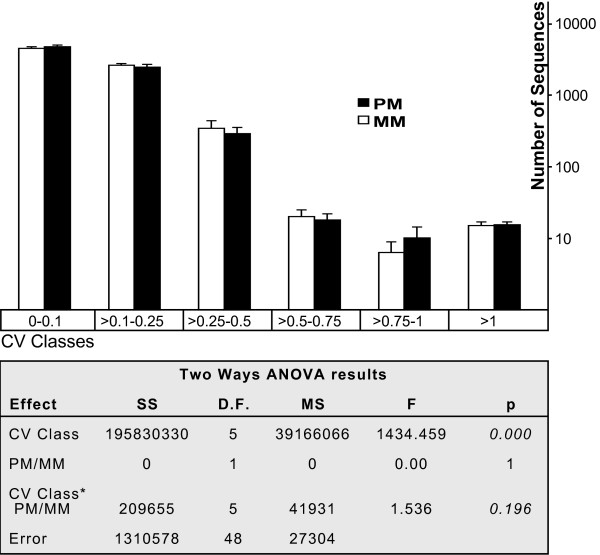
**Comparison of the number of MM and PM sequences *per *CV class in human samples**. All details and features of the figure are the same as in figure 1. The differences in the number of sequences *per *class between PM and MM sequences are analyzed by the interaction term (CV Class*PM/MM).

A two-way ANOVA was used initially to test whether or not all species presented a similar number of sequences in each of the following classes of decreasing reproducibility: CV<0.1, extremely reproducible; 0.1<CV<0.25, highly reproducible; 0.25<CV<0.5, reproducible; 0.5<CV<0.75, slightly reproducible; 0.75<CV<1, poorly reproducible; CV>1, very poorly reproducible. The number of sequences in these variability classes (Figure 1 and Additional file 5) significantly differed between species (interaction term CV Class*Species; F(20,96) = 1.99, p = 0.014). However, Tukey's post-hoc test showed that the differences were restricted to a greater number of sequences with CV below 0.1 in *Macaca fascicularis *than in *Homo sapiens *(p = 0.0109; Figure 1), a difference that may be considered irrelevant. Therefore, same-species analysis using human samples neither yielded more reproducible results than cross-species analyses of samples from other mammals nor differed in any relevant aspect. A comparison of human PM and MM data yielded similar results. No significant differences were observed in the number of sequences within each CV class, between both sets of data (interaction term CV class*PM/MM; F(5,48) = 1.536, p = 0.196; Figure 2), corroborating that sequence differences do not affect the distribution of the number of sequences in the defined hybridization variability classes.

### Identification of probe sequences yielding poorly reproducible hybridization values

**Figure 3:**
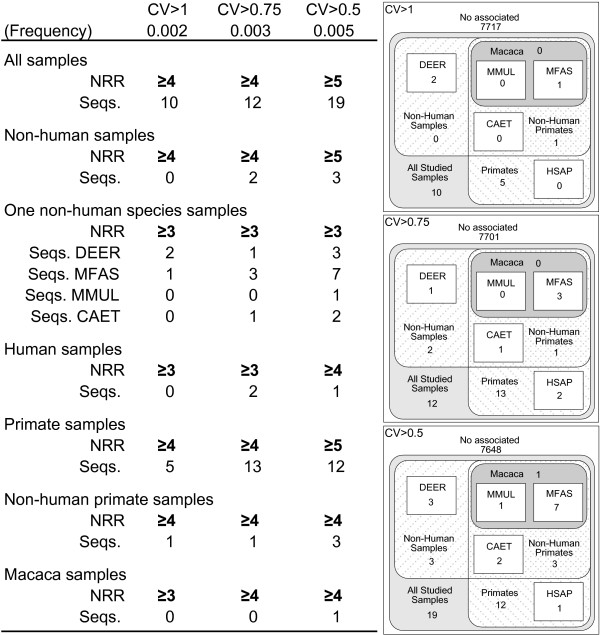
**PM un-reproducible probe sequences (UPS) for the different species or groups of species**. Results are detailed for each species or group of species and for CV over 0.5, 0.75 and 1. *Freq *details the probability of a sequence to present a CV over each boundary. *NRR *corresponds to non-random range and specifies the number of samples with a CV over a defined value (0.5, 0.75 and 1) in a given sequence that cannot result from a random distribution of the hybridization values in each species of group of species. S*eqs *specifies the number of sequences yielding poorly reproducible hybridization in each species or group of species. On the right side there is a graphic representation of the distribution of the UPS in the different species and groups of species. Detailed description of the calculi and the intermediate data can be obtained from the Additional file 6.

The next step in the study of the effects of cross-species analyses on reproducibility was the identification of probes that, due to features of their sequences or of their hybridizations with the transcript, resulted in high hybridization variability in any of the analysed species or groups of species and to evaluate whether their number changed in cross-species analyses. These probe sequences, called throughout the text "un-reproducible probe sequences" (UPS), were defined as sequences presenting high hybridization CV in more samples than could arise from a random combination of the CV values. UPS were identified for all the species studied and for groups of these species (all species, ALL; non-human species, N.H.S.; primates, PRIMATES; non-human primates, N.H.P.; and genus *Macaca*, MACACA). To identify UPSs for a species or group of species, we calculated the probability of a sequence to present high CV values (over 1, 0.75 or 0.5) in *n *samples of each species or group of species. The matrices detailing these probabilities are available in the Additional file 6 and summarized in Figure 3. For any given sequence, a probability below 0.01 was chosen to define the maximum number of samples than may randomly present a high CV (see Additional file 6). p < 0.01 was chosen because it is the most widely used significance limit. Other confidence limits will probably identify different probe sequences as UPS, but we are confident that they would not change the relative proportions of UPS in the different species or groups of species. We identified 19 sequences within this limit associated with CV>1, 35 with CV>0.75 and 52 with CV>0.5, in any of the species or groups of species defined, representing a fraction between 0.24 and 0.675 percent of the analysed sequences. Nearly half of these UPSs yielded un-reproducible hybridizations in both human and non-human species (Figure 3 and Additional file 7). Almost the same number of UPS was identified for primate (including human) samples, while just a few sequences showed poor reproducibility restricted to individual species or other groups of species. A description of all UPS (including probe sequence, Affy probe ID and Target according to Affymetrix and to BLASTn), as well as the identity of the group in which these sequences yielded high hybridization CVs, is available in the Additional file 8. Remarkably, many of the UPS hybridized to the same transcripts. As a consequence, while 52 sequences yielded un-reproducible hybridizations in any of the groups or species (associated to CV>0.5), they corresponded to only 32 targets. Six of them corresponded to non-human bacteria transcripts, spiked in the hybridization mixture to measure array performance, 11 to poorly defined human targets and the remaining 15 targets to clearly identified genes. The targets of the sequences yielding very poorly reproducible hybridizations (those with CV>1) in all samples were, in 8 out of 10 cases, non-human spike probes, while the remaining 2 sequences corresponded to poorly defined human targets.

**Figure 4:**
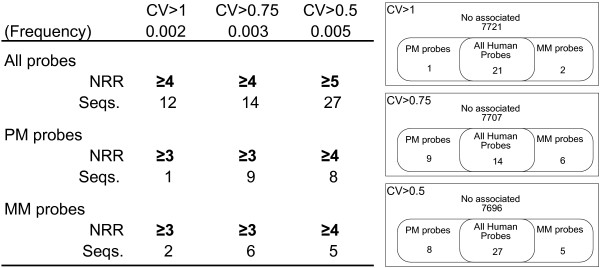
**PM and MM un-reproducible probe sequences (UPS) for human samples**. Results are detailed for MM and PM sequences, and for CV over 0.5, 0.75 and 1. All details as in figure 3.

PM sequences yielding un-reproducible hybridizations with human samples were also compared to the MM sequences yielding un-reproducible hybridizations with the same samples. These analyses showed that 15 sequences were associated to hybridizations with CV>1, 29 with CV>0.75, and 40 with CV over 0.5, either in PM or MM sequences, or in both at the same time (Figure 4 and Additional file 7). The total number of sequences identified as UPS in any of the three levels defined (CV>1, 0.75, or 0.5) rose to 43. Information on these sequences, including nucleotide sequence, Affy probe ID and Target according to Affymetrix and to BLASTn, as well as the identity of the group is shown in Additional file 8. Some of these sequences were repeated just once in the array, but others were used in up to 7 probes. In many cases, poorly reproducible MM sequences corresponded to poorly reproducible PM sequences (12 out of 15 with CV>1, 14 out of 29 with CV>0.75, and 27 out of 40 with CV>0.5). The number of PM or MM sequences associated to poorly reproducible hybridizations remained similar, indicating that sequence differences between PM and MM probes did not result in a different number of UPSs. Affymetrix and BLAST-derived annotations showed that the 43 identified UPSs hybridized with 31 different targets. The expression of some targets was evaluated after hybridization with up to 5 UPS. Of all identified sequences, 11 had non-human spike targets, whereas the remaining 32 corresponded to human mRNA, 14 of which had poorly defined targets.

### Effect of sequence differences on the values of the hybridization variability

**Figure 5:**
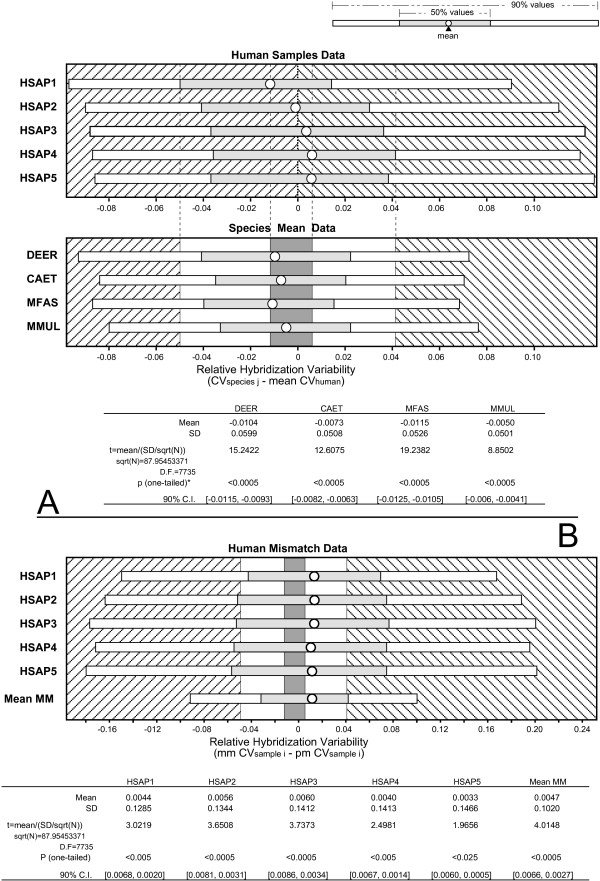
**Comparison of the hybridization variability in repeated sequences**. (a) Comparison between human and non-human PM data and (b) between PM and MM human data. Graphs detail the mean, interquartile and 50 to 95 percentile ranges and illustrate the magnitude and direction of each sample or species CV changes respect to the mean human PM CV. White and grey ranges in graphs "species mean data" and "human mismatch data" are defined after the human samples mean range (white area) and the interquartile range (grey area) and used to explore the magnitude of the change. Tables below each graph details the results of the one-tailed *t *test used to determine if the differences were significantly larger than 0. Sample codes as detailed in table 2. (*) denotes significantly different mean hybridization CV between a given non-human species and human.

A paired t-test was used to investigate whether or not hybridization variability of the individual PM sequences was significantly different when analysing non-human samples than when analysing human ones. We tested whether the difference in hybridization CV for every sequence in each species with respect to the human hybridization CVs (data matrices available in the Additional file 9) was significantly greater than 0. The unexpected results showed that all analysed non-human species yielded hybridization values significantly less variable i.e., more reproducible, than human hybridization values (Figure 5a). To examine how relevant these differences were, we compared them graphically to the 90% and 50% variability ranges of the human samples (Figure 5). Such comparison showed that the observed differences between human and non-human CVs were within the variation range of the human samples (Figure 5A). Moreover, the 50% and 90% ranges for the difference between the CV of the non-human species and the human CV were also located within the 50% and 90% ranges of the human samples. Taken together, these results indicate that hybridization of non-human samples to the human probes was not less reproducible than hybridization of human samples. The observed differences in reproducibility may be considered trivial when compared to the internal variation of the human hybridizations. However, a comparison of the human PM and MM hybridization variability yielded a totally different result. All MM and PM comparisons-either mean or individual sample data-show that MM hybridizations were significantly more variable (less reproducible) than PM ones (see Figure 5b). Moreover, a graphic comparison of MM mean values and ranges with the corresponding PM values shows that MM hybridization variability was in all cases undoubtedly greater than PM hybridization variability and well beyond the ranges defined by the human PM samples. Thus, it seems that the sequence differences of MM probes cause a significant and appreciable decrease in hybridization reproducibility with respect to the PM probes.

### Effect of hybridization level on reproducibility

**Figure 6:**
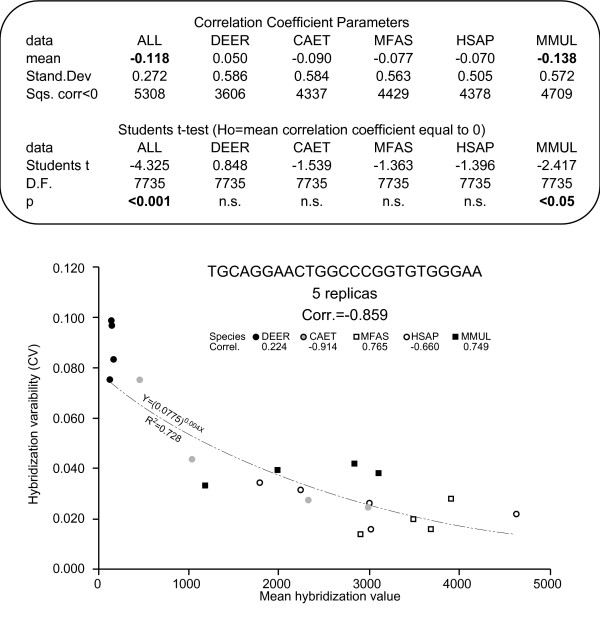
**Correlation between mean hybridization value and CV value in the repeated sequences**. Correlation coefficients were computed for each individual species and for all species together. The upper part of the table details the mean and standard deviation of the correlation coefficients of 7736 repeated sequences in each case. It also specifies the number of sequences with a negative correlation between mean and CV. Lower part of the table details the t-test parameters. The scatter plot below illustrates the correlation between mean hybridization value and CV value in a sequence repeated in 5 different probesets. It shows that correlation exists when all species are considered together but that it does not hold for the individual species.

According to previous authors [9,10,15,16], the most important negative effect of cross-species analyses is the reduction of microarray sensitivity, which, in turn, may change its reproducibility [15]. To determine whether or not hybridization signal and hybridization reproducibility are related, the correlation coefficient of mean hybridization values on hybridization CVs was calculated independently for the samples of each species and for all samples together. When all species samples were considered together, 5308 out of the 7736 repeated sequences (68.6%) presented a negative correlation between mean hybridization and hybridization CV, with mean correlation significantly below zero (p < 0.01, Student's t-test; see Figure 6). This relationship stands still even when deer data is excluded to avoid the effect that may result from its large leverage (t = -4,209, D.F.= 7735; p < 0.01). This indicates that if the samples for all species are considered together, the sequences present less reproducible hybridizations when their hybridization values are low. In contrast, analysis of individual species data yielded more variable results. Only one species, *Macaca mulatta*, agreed with the overall pattern, presenting a negative correlation in 4709 sequences (60.9%) and a mean correlation below zero, while the other primate species presented a more even distribution of the correlation values, with approximately 57% of the sequences showing negative correlations (4337, 56.1%; 4429, 57.3%; 4378, 56.6%) and a mean correlation that did not differ significantly from zero. *Cervus elaphus *samples presented a mean positive correlation, although not significantly different from zero (see Figure 6), with just 3606 sequences (46.6%) presenting negative correlation between hybridization mean and CV. Taken together, these data indicate that low hybridization values correlate with some decrease of reproducibility, although such relationship stood only for interspecies comparisons, not when comparisons were restricted to individual species.

## Discussion

We showed here that over 95% of the 7736 PM sequences repeated in different probe sets of the human U133 plus 2.0 GeneChip, yielded reproducible hybridizations in all the mammalian species analysed. Human and non-human samples presented a similar number of sequences in different reproducibility categories, except for a minor and irrelevant difference in the number of highly reproducible sequences between human and *Macaca fascicularis *samples. Moreover, hybridization of non-human RNA on human microarrays did not cause more sequences to be associated to un-reproducible hybridizations than did human RNA. Hybridization reproducibility was also similar across sequences, without relevant changes associated to cross-species analyses being observed. These results demonstrate that cross-species hybridizations of non-human mammalian samples to human U133 plus 2.0 GeneChip result in hybridization values that do not differ in reproducibility from the hybridization values of the human samples, even when analysing samples of species as phylogenetically distant to *Homo sapiens *as *Cervus elaphus*.

Lack of effects of cross-species on hybridization reproducibility points that sequence differences between the transcripts of the sample and the probes of the array do not result in changes in the reproducibility of the hybridization values with respect to fully complementary transcripts and probes. The reason underlying this result could be related, in our opinion, to the behaviour of the transcript-probe duplexes. It is well established that the stability of a transcript-probe duplex depends on the base sequence [26]. Under given physical and chemical hybridization conditions, any transcript-probe duplex will present a specific stability that may be estimated from knowledge of the number and identity of their complementary bases. Duplex stability determines the number of transcripts that will be hybridized to the probes forming a duplex. That is, for any given number and identity of the complementary bases between transcript and probe, there is a fixed probability for a transcript to be hybridized with the probe forming a duplex. Thus, a smaller number of complementary bases will result in a lower probability of transcript-probe hybridization reducing the hybridization signal, but not making it more variable. It is reasonable to think that even cross-hybridization of the probes with unspecific transcripts will follow this scheme. Thus, cross-hybridization will increase the noise of the hybridization value, but will not change the reproducibility of the data. All these reasoning explains why regardless of the mammal species being analyzed, reproducibility of the hybridization values remains almost constant. This is perfectly illustrated by our results from the deer analyses. Sequence differences between the deer samples and the human microarray cause a strong decrease in the hybridization reflected in the fact that, after scaling, less than 10% of the genes arrayed in the U133 plus 2.0 GeneChip are detected as present (according to MAS 5.0 algorithm, data not shown) but, as shown by our results, they do not cause any significant change in the reproducibility of the hybridization values.

Previous analysis of non-human primate and human gene expression with human GeneChips showed reproducibility differences between same-species and cross-species analyses [15,17]. These studies found that, although the number of genes that change their detection call (presence or absence of a transcript) across replicates (*flux genes *of Chismar *et al*. [15]) was similar, there was a clear difference in the distribution of the variability between same-species and cross-species analyses. Flux genes were more associated to higher signal intensities in cross-species analysis than in same-species ones [15]. As a result the number of genes consistently detected across replicates showed a significant decrease in cross-species analyses (from 24 to 12% according to Wang *et al*. [17]) and the size of the flux gene set as a percentage of the genes called present became clearly higher in cross-species analyses than in same-species analyses (27% vs. 17% in the analysis of [15]). The apparent contradiction of these observations and our results is probably due to the differences in the data employed. In the present study, we have used raw intensity values, whereas both Detection Call and Signal Intensity are gene expression measures that summarize the information on the probe level data after applying various analytical methods to the intensity values [27]. These analytical methods imply the use of several algorithms to normalize and quantify probe hybridization, followed by the application of other algorithms to summarize the information of the probes of each probeset into gene expression values. The analytical methods most commonly used, such as MAS 5.0 [28], dChip [29] or RMA (Robust Multi-chip Average [27]), differ in their approach and performance and seem to affect the reproducibility of the gene expression measures in both same-species and cross-species analyses [15,17]. In fact, the reproducibility of Detection Call and Signal Intensity appears to depend on the algorithm employed, with RMA and dChip algorithms yielding more reproducible results than the Affymetrix MAS5 [17]. Our study demonstrates, in this respect, that the reproducibility differences of gene expression measures seen in some previous cross-species analyses [15,17] were not due to differences in hybridization reproducibility. Rather, they were probably caused by the processing of the hybridization data, that is, by the algorithms used to estimate the presence and abundance of the transcripts. This conclusion may also explain why cDNA microarrays do not show any change in the reproducibility of their gene expression measures when performing cross-species analyses [8,19], while Affymetrix oligonucleotide microarrays do. Since cDNA microarrays use just one probe per target, gene expression measures are directly calculated from the intensity values of the probe, without using any algorithm that may affect the reproducibility of the estimates. It also could be that cDNA microarrays yield reproducible gene expression measures in cross-species analyses due to the use of long stretches of nucleotide sequence (up to a complete open reading frame), which would favour their specific hybridization with orthologous genes. However, as we have demonstrated, short oligonucleotide probes also result in reproducible hybridizations in cross-species analyses, in agreement with some authors that have proposed that results from individual oligonucleotide probes are as reproducible as those from long cDNA probes [8]. In fact, Agilent has been manufacturing oligonucleotide arrays for years with either three 25 bases long oligonucleotides per gene or one 60 bases long oligonucleotide per gene, resulting in hundreds of published papers [9].

The analysis of the relationship between hybridization value and coefficient of variation showed a negative correlation between both variables, i.e. high hybridization values were more reproducible than low hybridization values. This correlation was found significant only when the data from all species were analysed together, but not when the analyses were restricted to individual species. Thus, the correlation arises from the combination of hybridization values and reproducibilities for different species (Figure 6) because, for a given sequence, some species yield low and poorly reproducible hybridization values, while others yield high and very reproducible hybridizations. A similar relationship between hybridization level and reproducibility of gene expression measures was proposed by Chismar *et al*. [15] to explain the differences in reproducibility between same-species and cross-species analyses. However, we have shown that human same-species hybridizations do not differ in reproducibility from cross-species hybridizations. Moreover, all species yield similarly reproducible hybridizations despite the presence of large differences in the hybridization values of the different species (mean hybridization value in HSAP = 494.7, DEER = 264.6, CAET = 564.8, MFAS = 969.8, MMUL = 655.2). This raises the question of why species give similarly reproducible hybridizations in spite of their differences in hybridization values. Probably, the differences in hybridization values are not high enough to affect the reproducibility in a way that may be detected by our analyses. The effects of hybridization level on reproducibility would be appreciable only if hybridization values from the different samples were very different. That could be the case for individual probes, but it will never cause the reproducibility of cross-species data to differ from same-species data. Indeed, any comparison of the expression profiles between two or more samples should always pass through a process of scaling and normalization that would make the overall hybridization values comparable [13].

The comparisons of the human PM and MM hybridization data show that they differ neither in reproducibility category distribution nor in the number of sequences associated to poorly reproducible hybridization values. However, the hybridization values of MM sequences tend to be less reproducible than the hybridization values of their corresponding PM sequences. This result is probably related to the negative relationship between the hybridization value and the coefficient of variation previously discussed. Since, for each probe pair, MM values tend to be smaller than PM values (at least, in same-species hybridizations), their CV will tend to be slightly but systematically higher. However, MM data are difficult to interpret. Affymetrix GeneChips include MM sequences to quantify non-specific binding (i.e. hybridization with RNAs different from the target), a value that is directly or indirectly subtracted from the PM value in order to obtain the specific probe hybridization measure [13,28,30]. However, MM hybridization values, besides measuring non-specific binding, also measure hybridization with the target [27], though diminished because of the nucleotide sequence difference. Furthermore, the MM hybridization value may under or overestimate non-specific binding respect to the non-specific binding with the PM sequence. Whatever the causes for MM reproducibility reduction, it is clear that the uncertainties associated to MM hybridization affect the reproducibility of the derived gene expression measures. As a consequence, the analytical methods that use PM and MM hybridization values to quantify specific hybridization with the target, also combine the variability of PM and MM hybridization measures, probably reducing the reproducibility of the gene expression values obtained. Thus, algorithms like RMA that use only PM data will produce more reproducible expression measures in cross-species analyses than dCHip and MAS 5.0, that use both PM and MM data to measure gene expression, as shown by Wang *et al*. [17]. Quantification of probe hybridization from PM and MM intensity values seems to be the critical step in the calculation of the expression measures from raw intensity data, as concluded also by Fan *et al*. [31].

Our analyses the effect of cross-species hybridization on the number of un-reproducible probe sequences have allow us to identify various probes yielding un-reproducible hybridizations in all samples, regardless of the species of origin. Such probes produce hybridizations so variable that should be considered useless for gene expression analyses according to the criteria proposed by [19], and therefore, their data are best eliminated from further calculations. The presence of probes yielding un-reproducible hybridizations highlights the need for a deeper analysis of the probe features in order to improve the gene expression data obtained from the GeneChip analyses. Harbig *et al*. [32] reached a similar conclusion analysing the targets of individual probes of GeneChip U133 plus 2.0. They demonstrated that about 37% of the probe targets of the U133 plus 2.0 array needed to be redefined. All these results point that research in Affymetrix GeneChips should not only concentrate on the mathematics of intensity data processing by developing new algorithms, but also on the evaluation of the performance of the probes themselves.

## Conclusion

The results of the present study demonstrate that cross-species analyses do not significantly affect the reproducibility of the hybridization data from Affymetrix GeneChips, at least analysing RNA from a mammal species with another mammal species microarray. Differences in the reproducibility of gene expression measurements between same-species and cross-species analyses using Affymetrix microarrays are more likely caused by the analytical methods used to calculate the gene expression measurements from the hybridization data than by a reduction on the reproducibility of the hybridization data itself. These results, together with those from previous authors [9,15,17,18], indicate that Affymetrix GeneChips permit to obtain feasible hybridization data in cross-species analysis. However, they also make evident that choosing the appropriate algorithm to convert such hybridization data into gene expression measures is a key step in these analyses if we wish to preserve the quality of the obtained hybridization data. In this respect, the variability of the MM hybridization values observed in this study indicates that algorithms or analytical methods combining PM and MM hybridization values to quantify gene expression do result in a loss of reproducibility of the obtained gene expression measures. MM probes can be useful to identify and choose the most conserved probe sequences in cross-species analyses but they become a source of noise when quantifying gene expression. In the course of our analyses, we have also identified several probes yielding un-reproducible hybridizations. Hybridization data from these probes is so variable that should be considered useless for gene expression analyses and therefore eliminated from further calculations or even from the GeneChip. The presence of such un-reproducible probes stresses the need of a re-evaluation of the probes performance in Affymetrix GeneChips.

## Methods

### Data for primates

Human and non-human primate gene expression data comes from Dillman and Phillips study [18] and were downloaded from GEO database of the NCBI ([33], accession number GSE2634; last accessed in November, 2005). Available files include Affymetrix raw data (.DAT, .CEL, .EXP files) obtained after analysing with the Affymetrix U133 Plus 2.0 GeneChip^®^, RNA samples of whole blood from *Macaca fascicularis *(4 samples, named here *MFAS1 *to *4*, Table 2), *Chlorocebus aethiops *(4 samples, *CAET1 *to *4*), *Macaca mulatta *(4 samples, *MMUL1 *to *4*) and *Homo sapiens *(5 samples, *HSAP1 *to *5*).

### Acquisition of data for deer

Deer tissue samples come from biopsies of an 8 year-old male, kept at the Experimental Farm of the University of Castilla-La Mancha (Albacete, Spain). Samples correspond to the soft tissues overlying bone (epidermis, dermis, periostium and derivatives) at different positions: samples *DEER1 *and *DEER4 *are from the antler tip; sample *DEER2 *from the antler base or pedicle; and sample *DEER3 *from over the frontal bone of the skull. *DEER1*, *DEER2 *and *DEER3 *samples were harvested during the period of maximal antler growth (60 days after casting the previous antlers) while *DEER4 *was harvested at the end of the antlers' growing period (120 days after casting). To obtain the samples, the individual was kept in a hydraulic restrainer and anesthetized with a low-dose combination of xylazine (0.5 mg/kg of body weight; Calier, Barcelona, Spain) and ketamine (1 mg/kg BW; Imalgene 100, Menial, Lyon, France). After taking the samples, anaesthesia was reversed with yohimbine (0.25 mg/kg BW; Sigma-Aldrich, St. Louis, MO, USA). Samples of the antler tip (*DEER1 *and *DEER4*) were dissected using a sterile saw blade while *DEER2 *and *DEER3 *samples were taken using 4 mm. diameter biopsy punches (Stiefel, Madrid, Spain). All procedures were carried out by veterinaries and approved by the ethic committees of the Spanish Science Research Council and the Ministry of Environment. Samples were frozen in liquid nitrogen and kept at -80°C until their processing. Frozen tissues were crushed in a mortar cooled in liquid nitrogen. Total RNA was extracted using TRIzol reagent (Invitrogen Life Technologies, Carlsbad, CA, USA) and purified using the RNeasy kit (Qiagen, Valen, CA, USA). RNA quality was assessed by electrophoresis in 2% agarose gels (Invitrogen Life Technologies, Carlsbad, CA, USA), containing 0.5 μg/ml ethidium bromide (EtBr, Sigma-Aldrich, St. Louis, MO, USA). RNA preparation, hybridization, staining, and scanning of the GeneChip^® ^U133 Plus 2.0 was carried out by the Progenika Biopharma laboratories (Derio, Spain)[34], following Affymetrix protocols. According to these protocols, spike controls were added to the hybridization mixtures to ensure the correct hybridization, washing, developing and scanning of the GeneChips. CEL, .DAT, and .EXP files of all 4 samples were obtained and employed in the analyses. .CEL and .EXP files may be downloaded from the GEO database [33] under the accession number GSE4064.

### Hybridization data

Hybridization values for all PM and MM probes were obtained from the .CEL files using the PM and MM routines of Bioconductor's "Affy" package [27,35].

### Probes

Information on Affymetrix U133 Plus 2.0 GeneChip^® ^probes was downloaded from the Affymetrix website ([36] last accessed in November, 2005). The tab-delimited text file thus obtained, details the sequence, position, number, and probeset of all probes in the GeneChip^®^. Additional information about the targets of the probes was obtained from the Affymetrix database at NetAffx web page ([37] last accessed in March, 2006) and from the NCBI databases searched using the nucleotide-nucleotide BLAST (BLASTn [38]) program of the NCBI ([39] last accessed in March, 2006).

### Statistical analyses

All above information about each probe and their hybridization values with the different samples were exported to a FileMaker Pro 5.5 database (FileMaker Inc., Santa Clara, CA, USA). The search tool of FileMaker was employed to identify all the sequences repeated in more than 1 probe (caution recommended, because FileMaker Pro 5.5 employs only the first 21 characters to carry out the search and thus it may recognize as repeated, sequences that differ in their last 4 bases). Probe information and hybridization values of all repeated sequences were exported to an Excel spreadsheet (Microsoft, Redmond, WA, USA) to be formatted before its analysis with the Statistica 6.0 package (Statsoft Inc., Tulsa, OK, USA) (see Additional file 1). Normality of repeated hybridization values was assessed in the sequences repeated more than 6 times, using Shapiro-Wilk W, Kolmogorov Smirnoff and Lilliefors tests (Additional file 2; for comparison of the different methods; see [40] and Statistica help). For each repeated sequence in the GeneChip, Statistica was used to calculate the number of replicas and the mean, standard deviation and coefficient of variation (CV) of the hybridization values for each RNA sample (see Additional file 3). PM and MM data were treated as independent samples.

The variability of the repeated hybridization measures was used to estimate hybridization reproducibility. Different measurements may be used to describe the variability of single values on repeated trials [14,24,25], but according to Hopkins [14], within-subject standard deviation is the most appropriate one. However, when standard deviation depends on the size of the measure, the coefficient of variation (CV) should be employed instead. Because in our dataset, the standard deviation correlated significantly with the mean (Kendall Tau over 0.3, p < 0.05 in all samples, see Additional file 4), CV was chosen as a measure of hybridization variability among replicas in all the analyses. CV data of the repeated PM sequences were used to compare the reproducibility in same and cross-species analyses. To further test the effect of sequence differences on hybridization reproducibility, we also carried out the same analyses comparing CV data for repeated perfect match (PM) and mismatch (MM) sequences in the human samples. Description of the statistical procedures of the analyses is based in the comparison of the PM data from the different species. The comparisons of the human PM and MM hybridizations dataset were carried out in the same way unless specified.

To test whether cross-species hybridizations affected the distribution of the hybridization reproducibility, the number of sequences in each of the following classes of decreasing reproducibility was calculated: CV<0.1, extremely reproducible; 0.1<CV<0.25, highly reproducible; 0.25<CV<0.5, reproducible; 0.5<CV<0.75, slightly reproducible; 0.75<CV<1, poorly reproducible; CV>1, very poorly reproducible. Then, the number of sequences in each class across species (detailed in Additional file 5) was compared using a two-way ANOVA, followed by an unequal number of samples post-hoc Tukey's test. Species and CV classes were used as independent factors in the analysis.

Probe sequences associated to irreproducible or poorly reproducible hybridizations – "un-reproducible probe sequences" (UPS) – were defined as sequences presenting high hybridization CV in more samples of a species than could result from a random combination of the CV values. Three levels of variation (CV>1, >0.75 and >0.5) were set as the boundaries of poor reproducibility. The probability of a sequence presenting a CV>X in *n *samples randomly was calculated based on a binomial (Bernoulli) probability distribution. First, p_CV>X_, the probability that a sequence in one sample presented a CV>X was estimated as the mean number of sequences (across all samples) with CV>X divided by the total number of sequences (7736). p_CV>X _was then used to calculate the probability of a sequence to present a CV>X in *n *samples (p_sqy, CV>X, n_), using the equation:

p_sqy, CV>X, n _= C_m, n _[(p_CV>X_)^n^(1-p_CV>X_)^m-n^]

where C_m, n _represents the number of combinations of a set of *m *objects taken *n *at a time and is given by C_m, n_= [(n!)/(r!(n - r)!)], where *m *is the total number of samples in the species or group of species considered, and *n *the number of samples with CV>X. The probability that at least one of the 7736 sequences analysed presents *n *samples with CV>X (P_CV>X_) was calculated as: P_CV>X, n _= 1-(1-p_sqy, CV>X, n_)^7736^. The probability values obtained from these calculations were used to determine the number of samples that may randomly present a CV>X. A probability below 0.01 was chosen to define the maximum number of samples that may randomly present a CV>X. These calculations were made for all species (4 samples in each non-human species and 5 human samples), and for the groups of species that follow: all species (ALL), non-human species (N.H.S.), primates (PRIMATES), non-human primates (N.H.P.) and genus *Macaca *(MACACA). The maximum number of samples that may randomly present CV>X for each species or group of species was calculated for the three levels of CV defined and used to identify un-reproducible probe sequences for each species or group of species. However, a probe sequence may present high CVs in samples from different species or groups of species. To precise in which of the species or groups of species of those listed above is the sequence associated to poorly reproducible hybridizations, we have used the following criteria: i) a probe sequence will be considered un-reproducible for a given group, if that group shows more samples with CV>X than randomly expected (p < 0.01) and the sequence is also considered un-reproducible for all species within the group or for none of them; ii) a sequence is not considered un-reproducible for a group, if the sequence shows more samples with CV>X than randomly expected (p < 0.01) for the group but also for only one subgroup or species within that group. In such a case, the probe sequence is considered un-reproducible for that species or subgroup; finally, iii) when a sequence is considered un-reproducible for two or more species, so it is for the smallest group that contains those species. Although these criteria are not absolute and other criteria would result in different assignations, we are confident that the overall results will not vary significantly. Description of the targets of identified UPS was obtained from Affymetrix analysis site (NetAffx [37]) and from the GeneBank EST sequences using the nucleotide-nucleotide BLAST (BLASTn) from the NCBI BLAST webpage [39].

For the analysis of the effect of sequence differences on the values of the hybridization variability, the mean CV for every PM sequence in each species was first calculated, and the data used to determine the difference in hybridization CV for every sequence in each species with respect to the human hybridization CVs (data matrices available in the Additional file 9). One-tailed *t *test was employed to determine whether or not the differences between the CV for each species and the human CVs were significantly greater than 0. To examine how relevant these differences were, interquartile and 5 to 95 percentile ranges were calculated for the CV differences between every species and the human and were compared graphically to the differences between human sample CVs and the mean human CVs (Figure 5). When evaluating whether the hybridization variability of the MM sequences was significantly larger than the variability of their corresponding PM sequences, individual sample PM and MM data together with mean data were also compared.

The possible relation between hybridization variation and hybridization signal was explored by calculating the correlation between hybridization CV and mean hybridization value in all repeated sequences. The correlation coefficient between both variables was computed for the samples of each individual species and for all samples together. Student's t-test was used to check whether or not overall correlation coefficients in each case were significantly different from zero.

## Authors' contributions

MND conceived and designed the study, participated in the acquisition of the microarray data, carried out the statistical analyses, and was responsible for much of the writing. WPT participated in obtaining the deer data, helped in the statistical analyses and in drafting and revising the manuscript. MNS is the head of the laboratory; he contributed to the conception and design of the study, and was involved in the writing, the revision and the final approval of the manuscript. All authors read and approved the final version of this manuscript.

## Supplementary Material

Additional file 1 **Raw data used in this study**. This spreadsheet contains the description of all repeated probes in the U133 plus 2.0 GeneChip, including the probeset to which the probe belongs (first column, under the header *Probe Set ID*), the oligonucleotide sequence (*Sequence*), probe position in the GeneChip (*Probe x *and *Probe y*), a code number (*Number*) and the hybridization value of each sample for that probe. Hybridization values are given separately for human PM and MM probes. Click here for file

Additional file 2 **Normality test of the hybridization values in repeated sequences**. Results of the Shapiro-Wilk W test of normality on all sequences repeated more than 6 times in the U133 Plus 2.0 GeneChip. The detailed nucleotide sequences corresponding to the PM probes. MM probes differ just in a nucleotide in the 13th position. Significant differences from a normal distribution are given as * p < 0.1; ** p < 0.05; and *** p < 0.0012 (equivalent to p < 0.05 after application of Bonferroni correction). The spreadsheet also includes the frequency histograms of the hybridization values of sequence GACAAGGTCGAGACATTCCTGCGCA in 2 samples with a non-normal distribution (p < 0.0012) of the data. It can be appreciated that the main cause of non-normality may be attributed to the presence of one outlier in each set of values. Click here for file

Additional file 3 **Basic statistics of the hybridization data for the repeated sequences**. This spreadsheet contains the information about the mean (*mean*), standard deviation (*sd*), coefficient of variation (*cv*) and minimum (*min*) and maximum (*max*) hybridization values for all repeated sequences in each sample. Again, human values for PM and MM probes are detailed as independent samples. The first and second columns of this spreadsheet specify the sequence and the number of times (*N. Rps*) that it is present in the GeneChip. This large spreadsheet can be opened using Microsoft Excel. Click here for file

Additional file 4 **Correlation between mean and standard deviation of hybridization values**. Kendall Tau correlations between Standard Deviation and Mean of each sample, including MM probe data from the human samples. Scatterplots of mean (X axis) *versus *Standard Deviation (Y axis) for some samples are provided for illustration. Axes are drawn in logarithmic scale. Click here for file

Additional file 5 **Number of sequences in CV classes**. Data matrices describing the number of sequences in each of the CV classes defined in the text. Quantification is detailed for each sample grouped as in the analyses: the first matrix (columns A to D) includes the data used for the species comparison while the second matrix (columns F to I) provides the information for the comparison of human PM and MM sequences. Click here for file

Additional file 6 **UPS Probability tables**. This spreadsheet details all the data and mathematical operations used to calculate the probability of different numbers of samples presenting a CV over a given value (1, 0.75, and 0.5) when hybridizing with a probe sequence. Most values were calculated using Excel formulas, seen by double-clicking on the values. Probability values are given for each number of samples and for each of the groups used throughout the study. All symbols and abbreviations are defined in the spreadsheet. Click here for file

Additional file 7 **Identification of the UPS**. This spreadsheet contains three matrices identifying the un-reproducible probe sequences (UPS) identified in the different species and the samples in which they present CV>X and another three matrices describing the un-reproducible probe sequences (UPS) identified by analysing the CV values of PM and MM probes after hybridization of human samples. Each matrix corresponds to a different CV boundary, the first one to CV>1, the second to CV>0.75 and the third to CV>0.5. Each matrix specifies the nucleotide sequence in its first column, which samples present CV>X in the following columns, while the last columns detail the species or group of species (or kind of sequence PM/MM in the human samples) for which that sequence presents more samples with CV>X than could be randomly expected. The final column specifies the species or group of species (or kind of sequence) for which we have considered un-reproducible the probe sequence in the first column. Click here for file

Additional file 8 **Characterization of the UPS**. Spreadsheet containing two matrices, one describing the PM un-reproducible probe sequences for the different species or groups of species, and the other describing the MM and PM un-reproducible probe sequences for human samples. For each sequence, the matrix details in the nucleotide sequence, the species or groups in which the sequence behaves as an UPS at the three defined levels (CV>1, CV>0.75, and CV>0.5), the GeneChip probesets containing that sequence and the target of the sequence according to Affymetrix annotations and to a search of the GeneBank sequences using BLASTn tool (see Materials and Methods in the text). Click here for file

Additional file 9 **Differences Data Matrix**. This spreadsheet includes the data matrix used to compare the hybridization variability for each sequence between humans and non-human mammals. For each samples, the matrix gives: columns C to H, the mean hybridization CV of each sequence in each species (calculated from the sample data in Additional file 3); columns I to M, the difference between the hybridization CV of the human samples and the mean human hybridization CV; columns N to R, the difference between the mean hybridization CV of non-human species and mean human hybridization CV and the difference between the human PM and MM mean hybridization CV (*HSAPmm*); columns N to R, the difference between the MM hybridization CV and the PM hybridization CV for each human sample. Codes for species and samples, as given in Table 2 of the text. Click here for file
